# Low and very low birth weight in puppies: definitions, risk factors and survival in a large-scale population

**DOI:** 10.1186/s12917-020-02577-z

**Published:** 2020-09-24

**Authors:** Amélie Mugnier, Sylvie Chastant-Maillard, Hanna Mila, Faouzi Lyazrhi, Florine Guiraud, Achraf Adib-Lesaux, Virginie Gaillard, Claude Saegerman, Aurélien Grellet

**Affiliations:** 1 NeoCare, Université de Toulouse, ENVT, 23 Chemin des Capelles, Toulouse, France; 2 Biostatistiques, Université de Toulouse, ENVT, 23 Chemin des Capelles, Toulouse, France; 3 Royal Canin, 650 Avenue de la Petite Camargue, 30470 Aimargues, France; 4 UREAR-ULiège, FARAH Center, Faculté de Médecine Vétérinaire, Université de Liège, B42, Quartier Vallée 2, Avenue de Cureghem 7A, 4000 Liège, Belgium

**Keywords:** Canine, Puppy, Epidemiology, Risk factor, Neonatal mortality, Litter size, Birth weight

## Abstract

**Background:**

Neonatal mortality (over the first three weeks of life) is a major concern in canine breeding facilities as an economic and welfare issue. Since low birth weight (LBW) dramatically increases the risk of neonatal death, the risk factors of occurrence need to be identified together with the chances and determinants of survival of newborns at-risk.

**Results:**

Data from 4971 puppies from 10 breeds were analysed. Two birth weight thresholds regarding the risk of neonatal mortality were identified by breed, using respectively Receiver Operating Characteristics and Classification and Regression Tree method. Puppies were qualified as LBW and very low birth weight (VLBW) when their birth weight value was respectively between the two thresholds and lower than the two thresholds. Mortality rates were 4.2, 8.8 and 55.3%, in the normal, LBW and VLBW groups, accounting for 48.7, 47.9 and 3.4% of the included puppies, respectively. A separate binary logistic regression approach allowed to identify breed, gender and litter size as determinants of LBW. The increase in litter size and being a female were associated with a higher risk for LBW. Survival for LBW puppies was reduced in litters with at least one stillborn, compared to litters with no stillborn, and was also reduced when the dam was more than 6 years old. Concerning VLBW puppies, occurrence and survival were influenced by litter size. Surprisingly, the decrease in litter size was a risk factor for VLBW and also reduced their survival. The results of this study suggest that VLBW and LBW puppies are two distinct populations. Moreover, it indicates that events and factors affecting intrauterine growth (leading to birth weight reduction) also affect their ability to adapt to extrauterine life.

**Conclusion:**

These findings could help veterinarians and breeders to improve the management of their facility and more specifically of LBW puppies. Possible recommendations would be to only select for reproduction dams of optimal age and to pay particular attention to LBW puppies born in small litters. Further studies are required to understand the origin of LBW in dogs.

## Background

The mortality rate of puppies during the first two months after birth is around 15% of the total number of puppies born [1–3]. As puppy survival continues to be an important welfare and economic concern for dog breeders, further investigations are required to improve breeding facility management.

Low birth weight (LBW) is known to be an important risk factor for puppy mortality and morbidity during early life [4–6]. Newborns with LBW have lower energy reserves, and less vigour and are thus at a disadvantage when competing to suckle and obtain colostrum. This results in reduced passive immune transfer. Indeed, colostrum is crucial for newborn carnivores as it provides the nutrients and necessary immunoglobulins to kick-start their immune systems [7]. Body temperature after birth and the thermoregulatory capacity of LBWs are also lower than in heavier neonates [8–11]. These factors could explain the increased risk of mortality in LBW puppies.

A definition of LBW based on the birth weight threshold associated with an increased risk of neonatal mortality, has recently been proposed [6]. In this study, puppies were grouped according to birth weight into three categories: low, moderate and high risk of neonatal mortality.

A better understanding of the determinants of LBW and factors influencing survival is crucial. Intrauterine growth regulation is complex and many factors (maternal, foetal and placental) can affect foetal growth rate and, ultimately, birth weight [12–14]. Available data on factors influencing birth weight in canine species are sparse [15, 16]. Moreover, no studies have specifically examined the risk factors for LBW. The objective is not to maximize puppy birth weight but rather to help breeders to decrease the prevalence of LBW newborns.

The first aim of the present study was to define low and very low birth weight (LBW and VLBW) in different dog breeds. Secondly, to investigate the association between the occurrence of LBW or VLBW and different maternal and foetal factors. Finally, to explore the factors identified in each category of at-risk puppies (LBW and VLBW) that influence survival during the neonatal period.

## Results

### Population characteristics

**Fig. 1:**
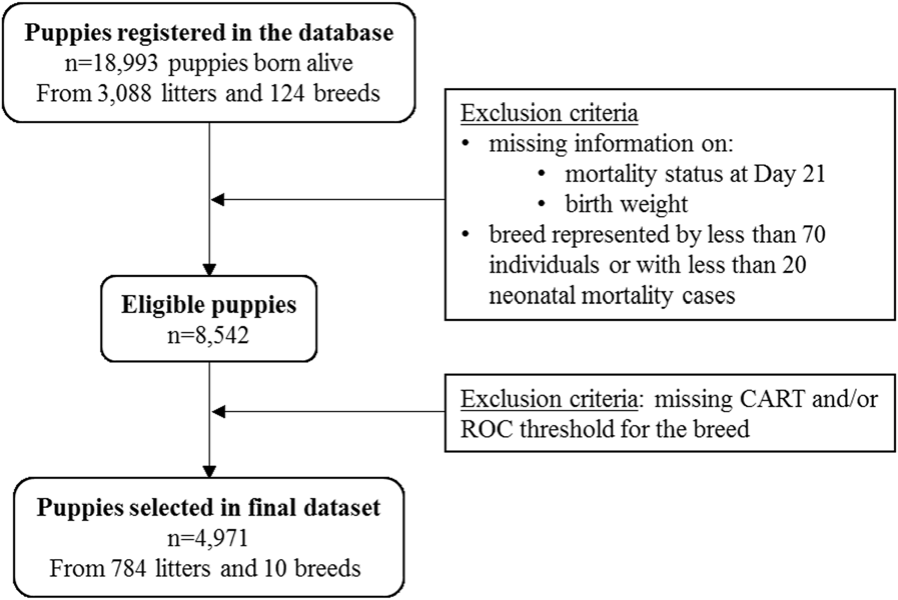
Flow diagram describing the data selection process

The population, after selection (Fig. 1), consisted of 4971 live-born puppies from 10 breeds, 784 litters, and 38 breeding kennels located all over France (Table 1). Litters were born between 1996 and 2019 with 76% of the litters born over the last 10 years (between 2009 and 2019). The median number of included puppies per breed was 275, ranging from 103 puppies for Bichon Frise to 1903 puppies for Labrador Retriever. Age of the dam at whelping varied from 1 to 9.9 years (median: 3.9; IQR, interquartile range: 2.7–5.3). The sex ratio was 1.0 (2498 males and 2460 females). Birth weights varied from 60 g (Bichon Frise) to 760 g (German Shepherd). The average within-breed birth weight ranged from 161.9 g (SD: 34.2) for Maltese to 513.7 g (SD: 90.8) for German Shepherd (Table 1). The global mean litter size was 7.1 puppies (SD: 2.6) and litter heterogeneity varied from 0 to 71.4% (median: 22.3%, IQR: 15.7–31.1). Among the 625 litters for which information was available, 193 contained at least one stillborn (31, 95%CI: 27–35). The overall neonatal mortality rate in the study population was 8.2% (406/4971; 95%CI: 7.4–9), with 36% (146/406; 95%CI: 31.3–40.8) of deaths occurring between birth and 2 days of age.

**Table 1:** Description of the studied population (*n* = 4971 puppies from 10 breeds)

Breed	Number of puppies included	% of the total population	Number of litters included	Mean birth weight, grams (± SD)	Mean litter size (± SD)	Mean litter heterogeneity, % (± SD)	Sex ratio	Litters with at least one stillborn, %	Neonatal mortality rate
Australian Shepherd	547	10.5	81	363.8 (±82.6)	7.6 (±2.1)	28.4 (±14.5)	1.1	27	5.5
Bichon Frise	103	2.0	20	180.1 (±35.6)	6.4 (±1.9)	25 (±15.9)	1.1	21	24.3
Cocker Spaniel	779	14.9	147	262.6 (±59.4)	5.7 (±1.9)	25.1 (±13.6)	1.0	23.9	6.5
German Shepherd	281	5.4	35	513.7 (±90.8)	7 (±2.9)	19.1 (±11)	1.0	35.7	7.8
Golden Retriever	588	11.2	79	394.8 (±76.8)	7.8 (±2.8)	25.4 (±12.7)	1.0	39.1	7.3
Labrador Retriever	1903	36.4	262	409.9 (±69.2)	7.8 (±2.7)	23.2 (±11.2)	0.9	33.8	6.2
Maltese	166	3.2	37	161.9 (±34.2)	5.1 (±1.8)	23.6 (±13.1)	1.2	22.9	15.7
Rottweiler	111	2.1	16	403.8 (±58.6)	8.2 (±2.4)	24.3 (±14)	1.4	37.5	18.9
Shih Tzu	270	5.2	58	175.5 (±27.9)	5.3 (±2.1)	17.8 (±9.4)	1.1	23.5	16.7
West Highland White Terrier	223	4.3	49	192.1 (±36.3)	5.3 (±1.6)	21.5 (±13.2)	1.1	28.6	11.2
Total	4971		784	350 (±116.2)	7.1 (±2.6)	23.7 (±12.7)	1.0	31	8.2

Neonatal mortality represents the number of puppies born alive but dying within the first 21 days of age divided by the number of born alive puppies. Sex ratio represents the ratio between males and females

### Determinants of low and very low birth weight

Among the 10 represented breeds, 48.7% (2419/4971; 95%CI: 47.3–50.1) of the puppies were of normal birth weight (NBW), 47.9% (2382/4971; 95%CI: 46.5–49.3) were in the low birth weight (LBW) category and 3.4% (170/4971; 95%CI: 2.9–4) in the very low birth weight (VLBW) category (Table 2).

**Table 2:** Distribution of the 4971 puppies included in the three birth weight categories

Breed	Number of puppies included	NBW			LBW			VLBW		
		Weight threshold	Number of puppies in this group (%)	Neonatal mortalityrate	Weight threshold	Number of puppies in this group (%)	Neonatal mortalityrate	Weight threshold	Number of puppies in this group (%)	Neonatal mortalityrate
Australian Shepherd	547	≥ 375	234 (42.8)	3.4	213–375	291 (53.2)	4.1	< 213	22 (4)	45.5
Bichon Frise	103	≥ 181	46 (44.7)	15.2	163.5–181	24 (23.3)	16.7	< 163.5	33 (32)	42.4
Cocker Spaniel	779	≥ 280	314 (40.3)	3.5	142.5–280	446 (57.3)	6.1	< 142.5	19 (2.4)	68.4
German Shepherd	281	≥ 480	184 (65.5)	3.3	338–480	90 (32.0)	12.2	< 338	7 (2.5)	71.4
Golden Retriever	588	≥ 417	223 (37.9)	4.5	177.5–417	359 (61.1)	8.1	< 177.5	6 (1)	66.7
Labrador Retriever	1903	≥ 406	1029 (54.1)	2.8	248–406	848 (44.6)	8.7	< 248	26 (1.4)	57.7
Maltese	166	≥ 163	85 (51.2)	8.2	115.5–163	60 (36.1)	11.7	< 115.5	21 (12.7)	57.1
Rottweiler	111	≥ 410	56 (50.5)	10.7	345–410	40 (36.0)	17.5	< 345	15 (13.5)	53.3
Shih Tzu	270	≥ 176	122 (45.2)	9.0	128.5–176	140 (51.9)	20.0	< 128.5	8 (3)	75.0
West Highland White Terrier	223	≥ 190	126 (56.5)	5.6	129–190	84 (37.7)	13.1	< 129	13 (5.8)	53.8
Total	4971		2419 (48.7)	4.2		2382 (47.9)	9	8.9	170 (3.4)	55.3

Neonatal mortality represents the number of puppies born alive and dying within the first 21 days of age divided by the number of born alive puppies. Weight thresholds are indicated in grams. NBW: normal birth weight; LBW: low birth weight; VLBW: very low birth weight

**Fig. 2:**
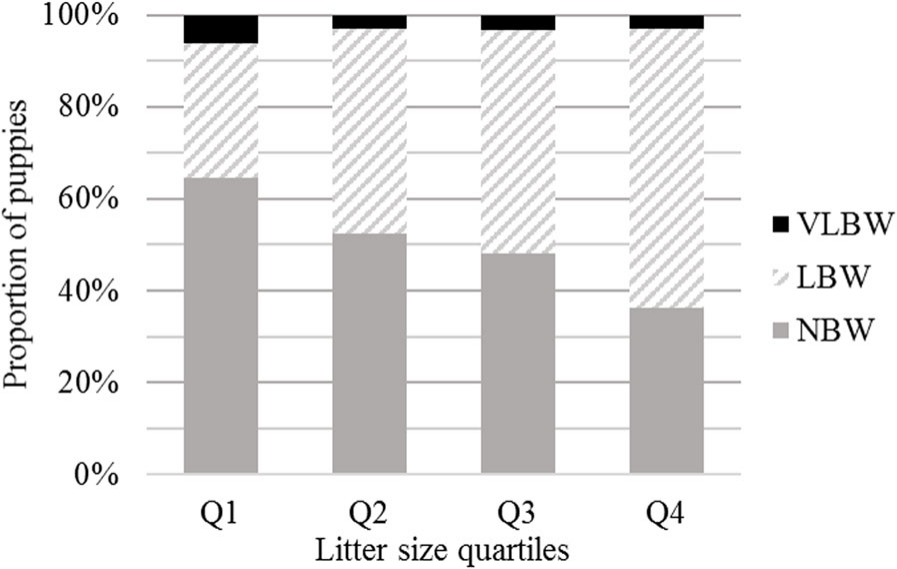
Distribution of puppies by birth weight category according to litter size quartiles. Q1 – puppies from litters with the lowest 25% of registered litter size values for the breed, Q2 and Q3–25% below and above the median, Q4 – puppies from litters with the highest 25% of registered values. NBW: normal birth weight; LBW: low birth weight; VLBW: very low birth weight

The results of the three binary logistic regressions are given in Table 3 together with the predictors included in the models, *P*, the proportional odds ratios and their corresponding confidence intervals. The risk of LBW or VLBW was significantly influenced by breed, litter size and gender. Depending on the breed, the proportion of LBW puppies varied from 23% in Bichon Frise to 61% in Golden Retriever and the proportion of VLBW varied from 1% (Golden Retriever) to 32% (Bichon Frise). Distribution of the puppies in the different birth weight categories was influenced by litter size (Fig. 2). The odds of having a LBW rather than NBW or VLBW, increased significantly with increasing litter size (OR = 8.1, 95%CI: 5.3–12.5 and OR = 14.5, 95%CI: 4.4–48.4, respectively). No difference was found when comparing litter size between NBW and VLBW puppies. The sex ratios were 1.2, 0.8 and 0.9 in the NBW, LBW and VLBW categories, respectively. The NBW category contained fewer females than the LBW category (45.1, 95%CI: 43.1–47.1 vs 53.8, 95%CI: 51.8–55.8 respectively; *P* < 0.001) but no statistically significant difference was found for the two other comparisons (LBW vs VLBW and NBW vs VLBW).

**Table 3:** Determinants of low and very low birth weight

Parameters	NBW vs VLBW [NBW as Reference]		LBW vs VLBW [VLBW as Reference]		NBW vs LBW [NBW as Reference]	
	*P*-value	Odds ratio (95%CI)	P-value	Odds ratio (95%CI)	P-value	Odds ratio (95%CI)
Dam age at whelping (years) [2–4 years as Reference]	0.081		0.536		–	
min-2		0.54 (0.18–1.33)		1.74 (0.7–5.12)		
4–6		0.99 (0.56–1.74)		1.23 (0.69–2.23)		
6-max		1.65 (0.88–3.06)		1.24 (0.62–2.58)		
Breed [German Shepherd as Reference]	< 0.001		< 0.001		< 0.001	
Australian Shepherd		0.98 (0.33–3.26)		2.53 (0.73–7.8)		1.93 (1.23–3.03)
Bichon Frise		8.27 (2.91–28.01)		0.11 (0.03–0.37)		0.92 (0.44–1.88)
Maltese		2.63 (0.83–9.35)		0.92 (0.23–3.42)		1.48 (0.82–2.66)
Cocker Spaniel		1 (0.33–3.37)		2.12 (0.6–6.71)		1.85 (1.17–2.93)
Golden Retriever		0.44 (0.11–1.68)		6.17 (1.54–25.29)		2.42 (1.55–3.79)
Labrador Retriever		0.33 (0.13–1.05)		5.01 (1.54–13.96)		1.4 (0.94–2.08)
Rottweiler		4.12 (1.35–14.27)		0.31 (0.08–1.02)		1.05 (0.56–1.99)
Shih Tzu		0.9 (0.17–3.91)		4.52 (1–23.91)		3.36 (1.94–5.88)
West Highland White Terrier		0.47 (0.07–2.22)		3.73 (0.7–27.73)		1.12 (0.64–1.98)
Litter size	–		< 0.001	14.47 (4.43–48.36)	< 0.001	8.11 (5.29–12.5)
Gender [Female as Reference]	0.063		–		< 0.001	
Male		0.65 (0.41–1.02)				0.69 (0.59–0.81)
Stillbirth in the litter [Absence as Reference]	–		–		0.47	
Presence						1.07 (0.9–1.27)

Two-by-two comparisons were conducted through multivariable binary logistic regressions. NBW: normal birth weight; LBW: low birth weight; VLBW: very low birth weight. Litter size is normalised by breed (formula: Y = (X – min) ÷ (max – min))

### Factors influencing survival of low and very low birth weight puppies

**Fig. 3:**
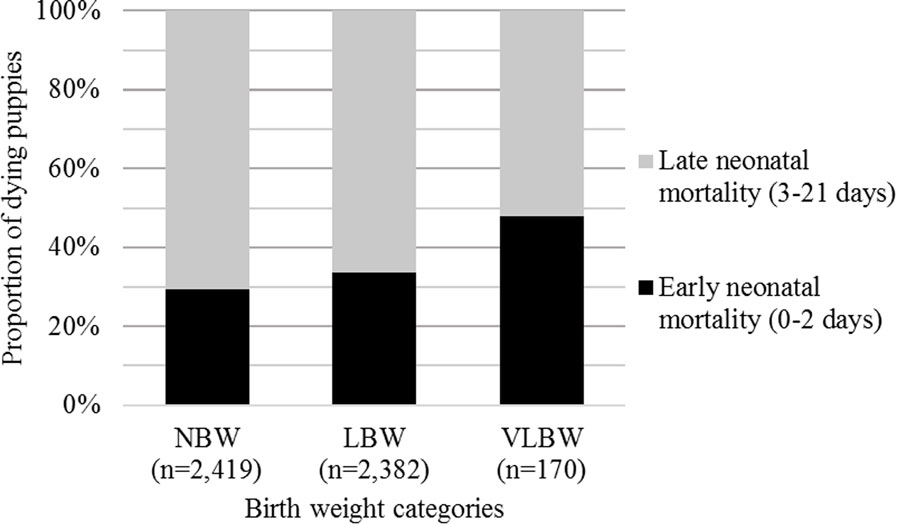
Repartition of the deaths between early and late neonatal periods by birth weight category. NBW: normal birth weight; LBW: low birth weight; VLBW: very low birth weight

Neonatal mortality rates were 4.2% for NBW puppies (102/2419; 95%CI: 3.5–5.1), 8.8% for LBW (210/2382; 95%CI: 7.7–10), and 55.3% for VLBW (94/170; 95%CI: 47.5–62.9). The proportion of deaths occurring between the early (0–2 days) and late (3–21 days) neonatal periods differed significantly according to the birth weight category (Fig. 3; chi-squared test, *P* = 0.027).

Application of the model to VLBW (*n* = 170) revealed that survival was influenced by litter size, but not gender. The odds of dying decreased significantly with the increasing litter size (OR = 0.05, 95%CI: 0.01–0.34; Table 4).

**Table 4:** Survival determinants in low and very low birth weight puppies

Parameters	VLBW [Survivor puppies as Reference]		LBW [Survivor puppies as Reference]	
	P-value	Odds ratio (95%CI)	P-value	Odds ratio (95%CI)
Dam age at whelping (years) [2–4 years as Reference]	–		0.001	
min-2				1.74 (0.87–3.36)
4–6				1.32 (0.81–2.15)
6-max				2.82 (1.66–4.8)
Breed [German Shepherd as Reference]	–		< 0.001	
Australian Shepherd				0.16 (0.04–0.57)
Bichon Frise				0.66 (0.12–2.94)
Maltese				0.65 (0.16–2.5)
Cocker Spaniel				0.42 (0.16–1.25)
Golden Retriever				0.46 (0.18–1.29)
Labrador Retriever				0.51 (0.22–1.32)
Rottweiler				1.37 (0.36–4.88)
Shih Tzu				1.82 (0.69–5.17)
West Highland White Terrier				0.83 (0.21–3)
Litter size	0.002	0.05 (0.01–0.34)	0.17	2.03 (0.74–5.66)
Gender [Female as Reference]	0.145		–	
Male		1.94 (0.8–4.87)		
Stillbirth in the litter [Absence as Reference]	–		0.005	
Presence				0.65 (0.41–0.99)

After selection of puppies from the considered category, two multivariate binary logistic regressions were conducted with mortality status at 21 days of age (dead/alive) as outcome. LBW: low birth weight; VLBW: very low birth weight. Litter size is normalised by breed (formula: Y = (X – min) ÷ (max – min)). When a parameter was not selected for the multivariate model after univariate analyses, a dash is provided in the P-value row

Application of the model to LBW (*n* = 2382) showed that survival was influenced by dam age at whelping, the presence of a stillborn in the litter and breed, but not by litter size (*P* = 0.001, *P* = 0.05, *P* < 0.001 and *P* = 0.17 respectively; Table 4). A significantly higher neonatal mortality rate was observed in puppies from dams more than 6 years old than in puppies from the reference category of dam age (16.3, 95%CI: 12.5–20.7 in ≥6 years vs 5.7, 95%CI: 4.3–7.3 in 2–4 year bitches). In litters with at least one stillborn, 7.8% (95%CI: 5.8–10.1) of the puppies died compared with 10.7% (95%CI: 9–12.5.) of the puppies in litters without any stillborn. As it is affected by breed, neonatal survival of LBW puppies varied from 95.9% (95%CI: 92.9–97.9) in Australian Shepherd to 80% (95%CI: 72.4–86.3) in Shih Tzu.

## Discussion

### Population

This work is the first national large-scale study on the risk factors of LBW in dogs. Seven of the ten canine breeds investigated belong to the top-twenty breeds born in France according to Société Centrale Canine (the French Kennel Club) [17]. The breeders included in the current study were widely distributed around France (Additional file 1). Even though the information collected in this study represented a large sample, a selection bias could not be excluded as the data were obtained via convenience sampling. Data collection was challenging due to the individual approaches of each breeder. Indeed, no professional tools centralising puppy birth weight data are currently available to French breeders. Having an automatic data collection system would increase the efficiency of future studies. Moreover, the final size of the dataset analysed (4971 puppies out of the 18,993 registered) underlines the challenge of dealing with missing data and working on various dog breeds (for many breeds, the number of puppies included was below the selected minimum threshold value of 70 puppies).

### Definition of low and very low birth weight

In this study, ROC and CART analyses were combined to define the birth weight thresholds within each breed and to identify puppies with an increased risk of neonatal mortality [9]. The puppies were therefore divided into three categories (NBW, LBW and VLBW) based on neonatal mortality and birth weight. Almost half of the puppies were below the ROC thresholds and were therefore classified as LBW, indicating that this category was not predictive enough. The CART thresholds made it possible to focus on the most critical population and almost 5% of the puppies were classified as VLBW.

### Determinants of low and very low birth weight

Multiple factors (placental, foetal and maternal) can cause intrauterine growth retardation, leading to low birth weight [14]. Some foetal and maternal factors were examined as potential risk factors of birth weight reduction in this study.

In many species, including dogs, males are consistently heavier at birth than females [15, 18, 19]. This could explain the larger proportion of females in the LBW category compared with the NBW category (Table 3). However, this difference was no longer observed when VLBW was compared with LBW or with NBW. This suggests that gender is not a risk factor for dramatic intrauterine growth retardation.

Previous studies of the influence of litter size on birth weight reported lower birth weights in large litters than in small litters [4, 5]. Litter size in the LBW category was significantly larger than in the NBW category (Table 3, Fig. 2). Surprisingly, VLBW was conversely significantly associated with smaller litter size, meaning that this drastic growth restriction cannot be explained by uterine crowding [20]. Further studies are needed to clarify the relationship between litter size and the occurrence of VLBW, and to identify potential causal factors in canine species.

### Factors influencing survival of low and very low birth weight puppies

The consequences of intrauterine growth restriction, including its relationship with neonatal mortality, have already been studied both in dogs and in other polytoccous species like pigs [6, 14, 21]. In the present study, birth weight had significant effects on neonatal mortality rate with 4.2, 8.8 and 55.3% for NBW, LBW and VLBW, respectively. Surprisingly, the LBW and VLBW groups presented different pattern of mortality: VLBW puppies died earlier than LBW with respectively 47.9 and 33.8% of puppies dying during the first two days of life (Fig. 3). This difference in mortality pattern suggests that the factors associated with survival in these two groups may be different, what was finally demonstrated by our statistical models.

Dam age at whelping had no significant influence on the occurrence of LBW or VLBW (Table 3) but had a significant effect on LBW survival (Table 4). In human medicine, several studies have reported an increased incidence of LBW in young mothers (less than 16 years) and in old mothers (more than 35 years old) who are more likely to present medical disorders during pregnancy or ante-partum [13, 22, 23]. In a systematic review conducted by Carolan and Frankowska, both mortality rate and the occurrence of LBW are increased for women aged 35–39 years [24]. Little is known about the relationship between advanced dam age and neonatal mortality in dogs [25]. Further research is warranted to explain this association and to define the optimal age for reproduction for each breed.

In this study, litter size influenced not only the occurrence of VLBW puppies but also their survival which was reduced in smaller litters. The factors affecting the occurrence of VLBW born in small litters might be the same as those explaining their reduced survival. For example, as in humans, placental insufficiency leading to foetal malnutrition might be one such factor [13]. It also could explain the reduced survival in puppies born under these conditions, due to possible abnormal development, including for example highly limited energy reserves. Future studies on canine placental characteristics and their relationships with puppy birth weight and survival could be interesting.

Not all the parameters that may influence the occurrence of LBW and VLBW and subsequent survival can be discussed here. Further investigations involving data collection for other factors associated with neonatal health and described in other species are required (e.g. placental insufficiency, maternal health issue and nutrition, parental body weights, environmental factors [13, 14]).

## Conclusions

Early detection of at-risk puppies with low birth weight is essential to reduce the neonatal mortality rate in canine species. This work confirms the necessity to conduct breed-specific analyses, with appropriate thresholds, when working on canine species. Litter size and dam age at whelping were found to be important factors affecting the occurrence of LBW or VLBW and subsequent survival. Particular attention needs to be paid to LBW and VLBW puppies born in small litters and/or from a dam more than 6 years old to ensure increased survival and thus improve animal welfare and kennel performance. Moreover, beyond the neonatal period, for surviving puppies, further research is required to study the mid-term and long-term consequences of reduced birth weight.

## Methods

### Data collection

The data used in this study were collected through a questionnaire (Additional file 2) sent out to French dog breeders from 2015 to 2019. The questionnaire was distributed using mailings, Facebook® messages, during canine exhibitions and via various dog breed associations [6]. Completion of the questionnaire was voluntary. Recorded data included information about the litter (date of birth, breed), dam (identity and age at whelping) and puppy (sex, birth weight, mortality during the first three weeks of life). This information was then anonymously transferred to a Microsoft Excel table (Microsoft Corporation, Redmond, Washington, USA) for analysis.

After data cleaning, all stillborn neonates, puppies with no birth weight provided or with unknown status regarding mortality and puppies from breeds with fewer than 70 individuals and/or with less than 20 deaths during the neonatal period, were excluded (Fig. 1).

### Variables definition

The remaining puppies were then classified into three categories, based on birth weight and status at 21 days of age (dead/alive), using two thresholds. One was determined by Receiver Operating Characteristics (ROC) analysis, and the other by Classification and Regression Tree (CART) analysis (detailed in [6]). Since the ROC threshold was systematically above the CART threshold, three categories were defined as follows: normal birth weight (NBW, puppies with birth weight at or above the ROC threshold), low birth weight (LBW, under ROC and above or at the CART threshold) and very low birth weight (VLBW, under the CART threshold). Puppies from breeds in which the CART or ROC analyses failed to identify any threshold were excluded.

The age (in years) of the dam at whelping was categorised into four groups: min-2; > 2–4; > 4–6; > 6-max, the reference category being “dam between 2 and 4 years old”. Litter heterogeneity represented the within-litter variation in birth weight and was expressed as the coefficient of variation (CV = standard deviation (SD) ÷ mean × 100 [10];). Significant associations were found between birth weight and breed, between litter heterogeneity and breed and between litter size and breed (all *P* < 0.001; Kruskal-Wallis rank sum test). Before analysis, these three parameters were transformed to allow between-breed comparisons. The birth weight values were normally distributed and were standardised by breed (formula: Y = (X – mean) ÷ SD). Litter size and litter heterogeneity were normalised by breed (formula: Y = (X – min) ÷ (max – min)).

### Statistical models

Three binary logistic regressions, allowing two-by-two comparisons, were applied to identify factors associated with the likelihood of belonging to a given category (NBW, LBW or VLBW). For each model, puppies belonging to the two categories involved in the paired comparison were extracted from the full dataset and their category represented the outcome (NBW vs LBW; LBW vs VLBW; NBW vs VLBW). The candidate explanatory variables were: dam age at whelping, litter size (total number of puppies born alive), stillborns in the litter (absence or presence), puppy sex and breed.

Factors related to the survival of LBW and VLBW puppies were analysed by constructing two binary logistic regressions. Puppies in the category under consideration (LBW or VLBW) were extracted from the full dataset and the binary variable (dead/alive) represented the outcome. The candidate explanatory variables were dam age at whelping, litter size, stillborn in the litter, sex, breed and litter heterogeneity.

For each model, preliminary bivariate analyses were applied to select candidate predictors (chi-square test of independence and Kruskall-Wallis rank sum test for the categorical and continuous variables, respectively). All candidate explanatory variables associated with the outcome with a *P* of 0.1 or less in the univariable analysis were included in the corresponding multivariable logistic regression [26].

The multivariable analyses were conducted by randomly dividing the selected subdataset into a training set and a testing set containing 70 and 30% of the data, respectively. The model was fitted on the training set and classification performances were evaluated on the testing set. The area under the ROC curve (AUROC) was then estimated to assess the ability of the model to differentiate puppies from the two considered categories (i.e. NBW vs LBW; LBW vs VLBW; NBW vs VLBW; dead vs survivor puppies). The appropriateness of the model was assessed by Pearson residuals test. This process was repeated 1000 times for each comparison and the results (*P* and odds ratio for each parameter, AUROC and *P* for the Pearson residuals test) were combined using the median.

Statistical analyses were performed with R version 3.4.2 [27] and the packages *pROC* and *rpart* [28, 29]. The level of statistical significance was set at *P* < 0.05 for all analyses. Statistical uncertainty was assessed by calculating 95% binomial confidence intervals (95%CI). The results from descriptive models were considered if the classification accuracy was acceptable (i.e. median AUROC over 0.65).

## Supplementary information

**Additional file 1.** Location of the breeding kennels included in the analysis.

**Additional file 2.** Survey.

## Data Availability

The dataset analysed during the current study is not publicly available due to issues of breeders’ confidentiality but is available from the corresponding author on reasonable request.

## Abbreviations

NBW: Normal birth weight
LBW: Low birth weight
VLBW: Very low birth weight
CI: Confidence interval
SD: Standard deviation
CV: Ccoefficient of variation
ROC: Receiver Operating Characteristics
CART: Classification and regression tree
IQR: Interquartile range
AUROC: Area under the ROC curve
OR: Odds ratio

## References

[1] Chastant-Maillard S, Guillemot C, Feugier A, Mariani C, Grellet A, Mila H (2017). Reproductive performance and pre-weaning mortality: preliminary analysis of 27,221 purebred female dogs and 204,537 puppies in France. Reprod Domest Anim.

[2] Indrebø A, Trangerud C, Moe L (2007). Canine neonatal mortality in four large dog breeds. Acta Vet Scand.

[3] Gill MA. Perinatal and late neonatal mortality in the dog. Doctoral dissertation. University of Sydney; 2001.

[4] Groppetti D, Ravasio G, Bronzo V, Pecile A (2015). The role of birth weight on litter size and mortality within 24h of life in purebred dogs: what aspects are involved?. Anim Reprod Sci.

[5] Mila H, Grellet A, Feugier A, Chastant-Maillard S (2015). Differential impact of birth weight and early growth on neonatal mortality in puppies. J Anim Sci.

[6] Mugnier A, Mila H, Guiraud F, Brévaux J, Lecarpentier M, Martinez C, Mariani C, Adib-Lesaux A, Chastant-Maillard S, Saegerman C, Grellet A (2019). Birth weight as a risk factor for neonatal mortality: breed-specific approach to identify at-risk puppies. Prev Vet Med.

[7] Mila H. Neonatal period in the dog: Immunological and nutritional determinants for survival. Doctoral dissertation. University of Toulouse; 2015.

[8] Cabrera RA, Lin X, Campbell JM, Moeser AJ, Odle J (2012). Influence of birth order, birth weight, colostrum and serum immunoglobulin G on neonatal piglet survival. J Anim Sci Biotechnol.

[9] Mila H, Grellet A, Delebarre M, Mariani C, Feugier A, Chastant-Maillard S (2017). Monitoring of the newborn dog and prediction of neonatal mortality. Prev Vet Med..

[10] Milligan BN, Fraser D, Kramer DL (2002). Within-litter birth weight variation in the domestic pig and its relation to pre-weaning survival, weight gain, and variation in weaning weights. Livest Prod Sci.

[11] Pedersen LJ, Berg P, Jørgensen G, Andersen IL (2011). Neonatal piglet traits of importance for survival in crates and indoor pens. J Anim Sci.

[12] Sankaran S, Kyle PM (2009). Aetiology and pathogenesis of IUGR. Best Pract Res Clin Obstet Gynaecol.

[13] Sharma D, Shastri S, Sharma P. Intrauterine growth restriction: antenatal and postnatal aspects. Clin Med Insights Pediatr. 2006. 10.4137/CMPed.S40070. 10.4137/CMPed.S40070 PMC4946587 27441006

[14] Wu G, Bazer FW, Wallace JM, Spencer TE (2006). Intrauterine growth retardation: implications for the animal sciences. J Anim Sci.

[15] Groppetti D, Pecile A, Palestrini C, Marelli S, Boracchi P (2017). A national census of birth weight in purebred dogs in Italy. Animals..

[16] Schelling C, Gaillard C, Russenberger J, Moseley L, Dolf G (2019). Heritabilities for the puppy weight at birth in Labrador retrievers. BMC Vet Res.

[17] Société Centrale Canine: les statistiques du LOF depuis 1969. https://www.centrale-canine.fr/articles/le-lof. Accessed 14 Aug 2018.

[18] Assan N (2013). Various factors influencing birth weight in animal production. Sci J Rev.

[19] Čechová M (2006). Analysis of some factors influencing the birth weight of piglets. J Anim Sci.

[20] Foxcroft GR, Dixon WT, Novak S, Putman CT, Town SC, Vinsky MDA (2006). The biological basis for prenatal programming of postnatal performance in pigs. J Anim Sci.

[21] Fix JS. Relationship of piglet birth weight with growth, efficiency, composition, and mortality. Doctoral dissertation. University of North Carolina; 2010.

[22] Valero de Bernabé J, Soriano T, Albaladejo R, Juarranz M, Calle ME, Martinez D, Dominguez-Rojas V (2004). Risk factors for low birth weight: a review. Eur J Obstet Gynecol Reprod Biol.

[23] Jolly M (2000). The risks associated with pregnancy in women aged 35 years or older. Hum Reprod.

[24] Carolan M, Frankowska D (2011). Advanced maternal age and adverse perinatal outcome: a review of the evidence. Midwifery..

[25] Schrack J, Dolf G, Reichler IM, Schelling C (2017). Factors influencing litter size and puppy losses in the Entlebucher Mountain dog. Theriogenology..

[26] Bursac Z, Gauss CH, Williams DK, Hosmer DW (2008). Purposeful selection of variables in logistic regression. Source Code Biol Med.

[27] R Core Team (2016). R: A language and environment for statistical computing.

[28] Robin X, Turck N, Hainard A, Tiberti N, Lisacek F, Sanchez J-C, Müller M (2011). pROC: an open-source package for R and S+ to analyze and compare ROC curves. BMC Bioinformatics.

[29] Therneau T, Atkinson B, Ripley B. rpart: Recursive Partitioning. R package version 4.1-3. 2013.

