# Supplementary material for: Low and very low birth weight in puppies: definitions, risk factors and survival in a large-scale population
Source: BMC Vet Res. 2020 Sep 24;16:354. doi: 10.1186/s12917-020-02577-z (PMC7517789; doi:10.1186/s12917-020-02577-z)

**Additional file 1.** Location of the breeding kennels included in the analysis. Postal code was missing for two of the 38 breeders who participated in the study (additional file created with ©BatchGeo LLC)


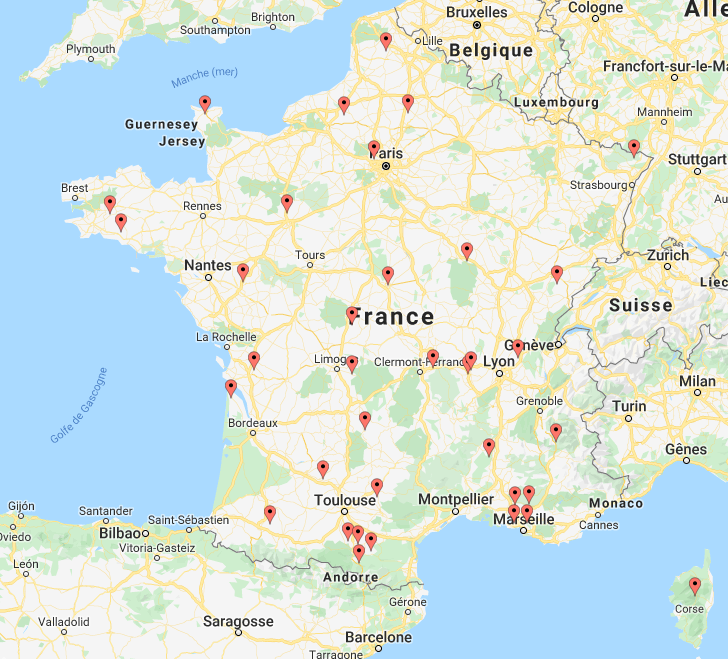

Supplement: Supplementary file 1 — Additional file 1. Location of the breeding kennels included in the analysis. [file 12917_2020_2577_MOESM1_ESM.docx]
