# Supplementary material for: Low and very low birth weight in puppies: definitions, risk factors and survival in a large-scale population
Source: BMC Vet Res. 2020 Sep 24;16:354. doi: 10.1186/s12917-020-02577-z (PMC7517789; doi:10.1186/s12917-020-02577-z)
Supplement: Supplementary file 2 — Additional file 2. Survey. [file 12917_2020_2577_MOESM2_ESM.docx]

**Survey on neonatal health in pure breed puppies**

Thank you for completing **one survey per litter**

In case you don’t have some information, please leave an answer blank.

**Your kennel**

In which country is situated your kennel?

**Female**

Name:       Breed:

Date of birth:

**Puppies**

Date of whelping (the first puppy born):

Total number of puppies born

Total number of stillborn puppies (dead at birth)


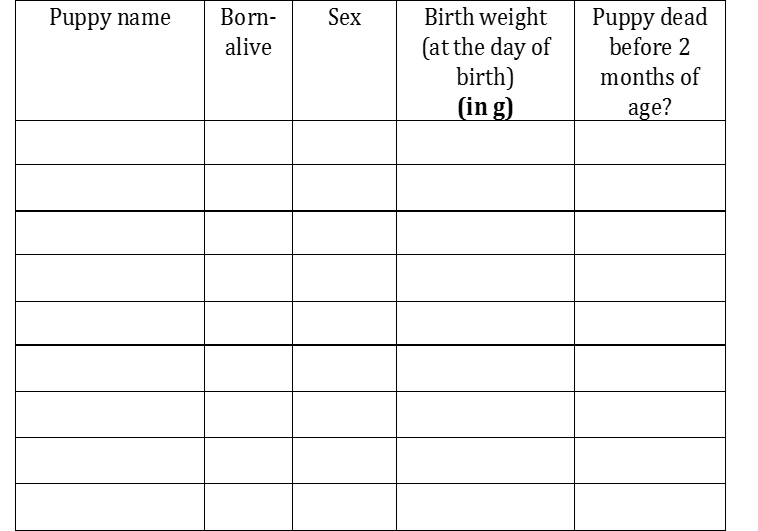
Total number of puppies born alive
